# Genome-wide identification and expression analysis of E2 ubiquitin-conjugating enzymes in tomato

**DOI:** 10.1038/s41598-017-09121-4

**Published:** 2017-08-17

**Authors:** Bhaskar Sharma, Tarun Kumar Bhatt

**Affiliations:** Department of Biotechnology, Central University of Rajasthan, Bandarsindri, Ajmer 305817 India

## Abstract

The ubiquitin-proteasomal degradation mechanism has gained the attention over the past decade. The E2 ubiquitin conjugating enzymes are the crucial part of ubiquitination mechanism and they are believed to hold imperative association for plant development. It accepts ubiquitin from the E1 enzyme and interacts with the E3 ligase to transfer ubiquitin or directly transfers ubiquitin to the substrate. The functional aspects of E2 ubiquitin enzymes in plant systems are unclear. Tomato is being used as a model plant and rarely explored to study E2 ubiquitin enzyme. We have utilized *in*-*silico* methods to analyze E2 enzymes in *Solanum lycopersicum* and 59 genes were identified with UBC family domains. The physio-chemical properties, chromosomal localization, structural organization, gene duplication, promoter analysis, gene ontology and conserved motifs were investigated along with phylogenetic analysis of tomato E2 genes exploring evolutionary relations. The gene expression analysis of RNA sequencing data revealed expression profile of tomato E2 genes in seedling, root, leaf, seed, fruit, and flower tissues. Our study aid in the understanding of distribution, expansion, evolutionary relation and probable participation in plant biological processes of tomato E2 enzymes that will facilitate strong base for future research on ubiquitin-mediated regulations in tomato and other plant systems.

## Introduction

Ubiquitin is 76 amino acid protein which binds to its target via lysine residue followed by ubiquitination and clearance of target through 26S proteasomal complex, thereby, control protein load in the cells^1^. The ubiquitination is a complex process, which involves three enzymes namely, E1 ubiquitin-activating enzyme, E2 ubiquitin-conjugating enzyme and E3 ubiquitin ligase. E1 activating enzyme initiates the ubiquitin-mediated degradation by adenylation at C-terminal of ubiquitin protein through an ATP-dependent reaction^2^. The cysteine residue in E1 activating enzyme, attacks ubiquitin, and generates E1-ubiquitin thioester intermediate. E1 activating enzyme transfers the ubiquitin molecule to E2 enzyme at conserved active site cysteine residue in the catalytic pocket of E2. The E2 conjugating enzyme catalyzes the transfer of ubiquitin to lysine on the substrate, forming an isopeptide bond along with E3 ubiquitin ligase enzyme. The E2 ubiquitin conjugating enzyme can also transfer the ubiquitin protein to catalytic cysteine residue to E3 ubiquitin ligase which ultimately mediates the transfer of ubiquitin to a lysyl group of substrate^2, 3^. E3 ligase recognizes the substrate and form ubiquitin thioester bond, then transfers the ubiquitin to a substrate^4^. The 26S proteasomal degradation complex, which destroys the ubiquitinated proteins, is made up of the 19S regulatory particle (RP) and the 20S core protease. It is responsible for degradation of the target protein. The E2 ubiquitin-conjugating enzymes are major determinants for selection of the lysine to construct ubiquitin chains, thereby targeting substrates. E2 enzymes posses highly conserved UBC domain of around 150 amino acids with cysteine residue. It has several binding sites for ubiquitin substrates and E3 ligase enzyme but only one active site^5^. The UBC domain consists of an anti-parallel β-sheet, a short 310–helix and four α-helices^6, 7^. The active-site cysteine (Cys) is located in a shallow groove between helix-2 and helix-3^8^. The previous reports on the identification of E2 enzymes members are 48 in rice^9^, 75 in maize^10^, 37 in *Arabidopsis thaliana* ^11^, 20 in *Caenorhabditis elegans* ^12^, 37 in human^5^, 13 in *Saccharomyces cerevisiae* ^13^, and 72 in banana^14^. The evolution of eukaryotic genome is predicted to be associated with a number of E3 ligase enzymes and DUB^15^ but as evident by previous reports^11, 16^, extension of E2 ubiquitin conjugating enzymes are also seem to be linked with genome evolution. It is observed that acquiring new molecular functions is the major factor for biological diversity^17^ and therefore, genes diversity leads to the altered functions of the original mechanism. There can be single or multiple changes within the sequences, arising to altered protein with similar functioning over the environmental influences due to gene duplication, which is a major factor of the expansion and divergence of gene family members^18^. Tomato is a popular model plant, especially, for the study of fruit development. Tomato has unique features compared to other model plants that make it as an alternative system for research. The assessment of E2 conjugating enzymes in tomato will help to explore mechanism and integrity of ubiquitin mediated proteasomal degradation in tomato and plant system. Micro-Tom cultivar of tomato was recognized as a model system for study as it has a short life cycle, small size and capacity to grow under fluorescent light^19^. RNA sequencing data of Heinz 1706 cultivar is available on Sol Genomics Network. Tomato plant cultivation is limited by pathogen attack, therefore, plant pathogen interaction study in tomato is required for developing efficient approaches to improve yield^20^. The E2 enzymes are reported to involve in both biotic and abiotic stress^21, 22^. A recent study showed that the tomato E2 conjugating enzymes were positively regulating immunity^23^. The E2 enzyme was also found to be involved in osmotic stress tolerance^24^, drought tolerance^25, 26^, and salt tolerance^27^. Further characterization of E2 gene family may provide the fundamental understanding of the distribution of ubiquitin-proteasome system and their action mechanism as it affects plant development and responses to the external environment. In the present study, we have identified and characterized the E2 enzymes in tomato genome and also, discussed expression profile during various conditions in different parts of the plant.

## Results

### Identification of E2 Ubiquitin Conjugating Enzymes in tomato

The UBC (Ubiquitin Conjugating) domain was retrieved from Pfam^28^ to generate HMM (Hidden Markov Model) profile in HMMER 3.0 package. HMM analysis is standard pairwise comparison methods for large-scale sequence analysis and is a method for searching homologous sequence by converting multiple sequence alignment into position specific scoring system^29^. We have identified 59 putative candidates by searching the generated HMM profile with default parameters and significant e-value of 0.01 against *Solanum lycopersicum* genomic sequence database (Taxonomy ID: 4081). The information of locus id, molecular weight, iso-electric point, length of amino acids, aliphatic index, instability index, grand average of hydropathy, exons, introns and sub-cellular localization were analyzed (Supplementary Table S1). The number of amino acids varied from 80 to 925 among the sequences. Most of the proteins identified were unstable according to instability index measure and hydrophilic in nature. The molecular weight of the proteins ranges from 9001.33 to 102829.66 Daltons with pI range of 4.41 to 8.93. Eight proteins were located in mitochondria, and four proteins were predicted in the cytosol and secretory pathways. It suggests that E2 enzymes are organelle-specific but regulated in variable tissue microenvironment.

### Chromosomal localisation and Phylogenetic analysis

**Figure 1:**
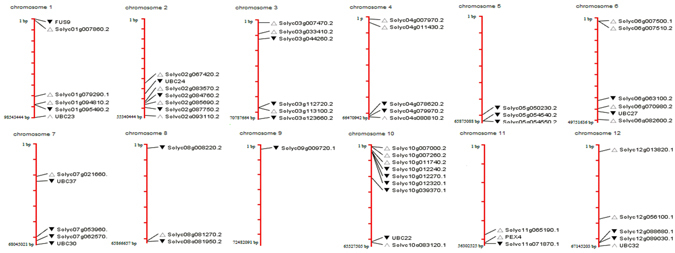
The diagram represents chromosomal map of tomato genome constructed by ArkMap software. All 59 E2 enzymes of tomato are localized on 12 chromosomes.

**Figure 2:**
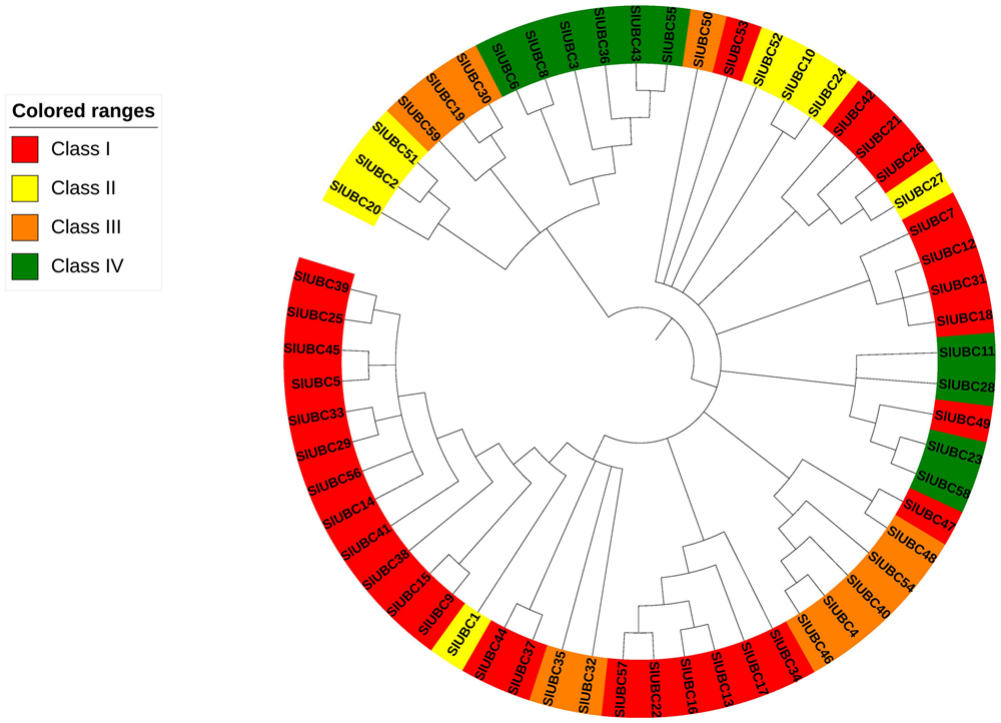
The phylogenetic tree of the *Solanum lycopersicum* E2 members is constructed by Neighbour-Joining method with 1000 bootstrap values. The E2 enzymes are divided into four classes and represented by different colors.

The E2 genes were distributed across all 12 chromosomes of tomato genome (Fig. 1). The highest numbers of genes were present on chromosome 10. Most of the genes were located on distal regions of chromosomes. Only one gene was located on chromosome 9. Using neighbour joining method, a phylogenetic tree was constructed to investigate evolutionary relationship and functional divergence among E2 ubiquitin-conjugating enzymes of *Solanum lycopersicum* with 1000 bootstrap replicates (Fig. 2). The 59 tomato E2 candidates were named SlUBC1 to SlUBC59 according to their position on chromosome (Supplementary Table S1). The phylogenetic analysis revealed the similarity among tomato E2 genes containing UBCc (Ubiquitin Conjugating Enzyme, E2 catalytic) superfamily domain and distributed into four groups according to presence of the only UBC catalytic domain (Class I), N-terminal extension (Class II), C-terminal extension (Class III) and both N- and C- terminal extensions (Class IV)^30, 31^ (Supplementary Figure S2). We found 30 E2 members belonged to class I and 8, 11, and 10 members belonged to class II, III and IV, respectively. The SlUBC14, 56, 29, 33, 5, 45, 25, 39, 41, 9, 15 and 38 were highly similar and categorized as largest a group and into class I. SlUBC19, 30 and 59 harboured trans-membrane domain at C-terminal, showed high similarity and categorized into class III. Except SlUBC32, no any E2 member in tomato has predicted ubiquitin associated (UBA) domain at its C-terminus. SlUBC2, 51 and 20 of class II and SlUBC6, 8, 3, 36, 43 and 55 of class IV were among closest E2 members. The observation of phylogenetic tree, showing great extent of similarity among tomato E2 members, indicates the expansion of E2 enzyme gene family in tomato, probably, through gene duplication and alternative splicing and predicted to evolve according to host specific requirements. Thus, tomato E2 enzymes represent a set of proteins with variation leading to unalike molecular functions.

### Motif and Gene structure analysis

**Figure 3:**
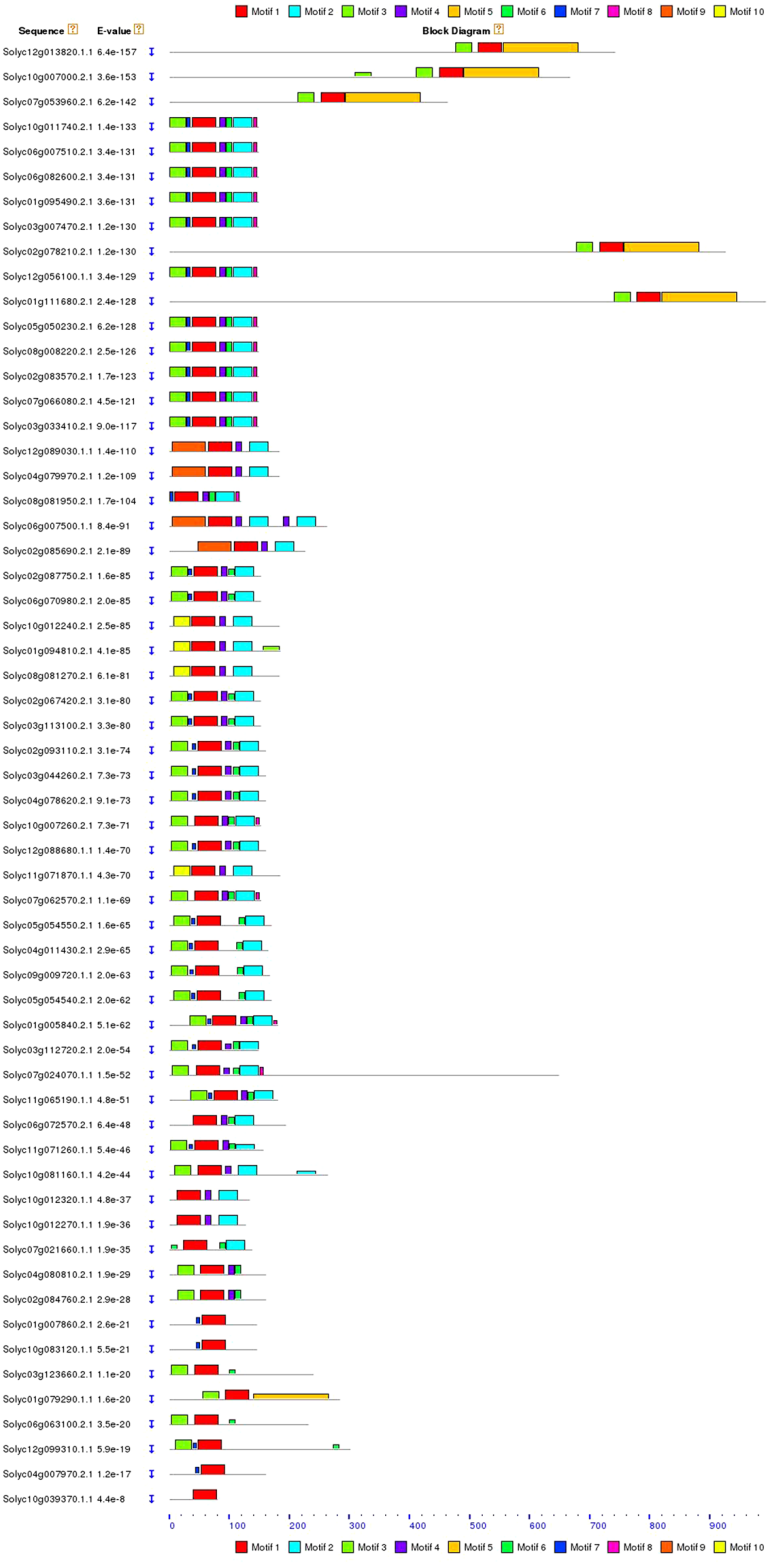
The discovered conserved motifs in all 59 sequences of tomato E2 enzymes are illustrated. A total of 10 motifs were discovered and their organization on the protein is represented by color boxes.

**Figure 4:**
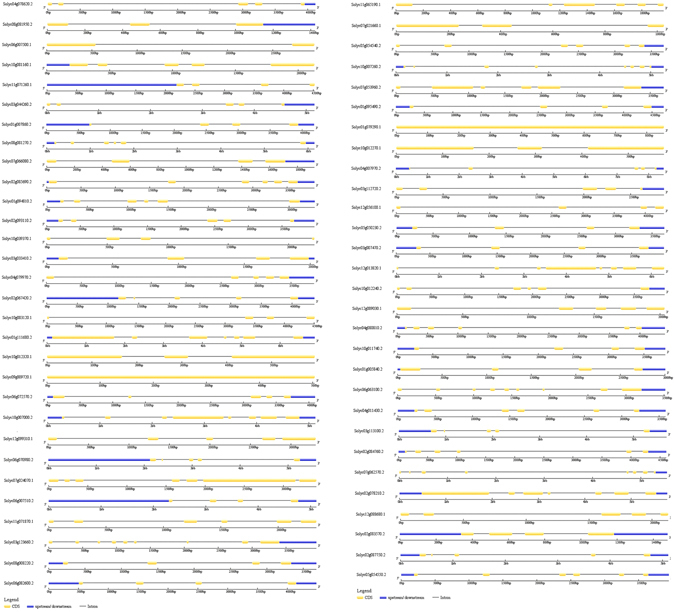
The exon/intron distribution of corresponding 59 identified tomato E2 enzymes was detected by comparing coding sequences (CDS) with their corresponding genomic sequences using GSDS tool online. The green box represents CDS; the blue boxes indicate upstream or downstream; the discontinuous lines refer to introns.

Ten different motifs were discovered for tomato 59 E2 members using MEME suite (http://meme-suite.org/) (Fig. 3). These are set of short sequences and are presumed to share biological functions. The motifs 1, 2, 3, 4, 6 and 7 were studded in most of the sequences which suggests the functional similarity and conserved position of the genes in tomato. The range of motif width was 8 to 126 amino acids (Supplementary Table S3). The motif 1 was the most conserved in all E2 members and motif 2 and 3 were present on C- and N- terminal, respectively. The motif 5, 8, 9 and 10 were selectively present in few E2 members which may serve additional or specific function. The complex structures with multiple domains predict their tight regulation pattern. The identified 10 motifs were scanned in human and Arabidopsis E2 members to evaluate the similarity (Supplementary Figure S4). We found that the motif 1 was present in all human, Arabidopsis and tomato E2 members and the motif 2, 3, and 4 were present in most of the sequences at almost similar pattern. The motif 5, 8, 9 and 10 were rarely found in human E2 members whereas Arabidopsis E2 members shared the major pattern of motif distribution with tomato. The distribution of motifs in tomato E2 members was more complex than in Arabidopsis. It indicates that the regulation of E2 members in tomato is more complex than human and Arabidopsis. The Arabidopsis E2 member At3g15355.1 shared motif 1, 3 and 5 and was found to be similar with SlUBC6 and 8 in tomato. The analysis of motifs found that motif 1 and 4 were crucial for the ubiquitin conjugating activity of the E2 enzymes and discovered motifs share significant structural and functional similarity with other organisms and predicted to participate in ubiquitin conjugation activity leading to the ubiquitination. The 59 identified E2 genomic DNA and complementary DNA sequences were aligned and structures were confirmed (Fig. 4). The maximum of 8 introns per gene to the minimum of zero intron per gene were identified in E2 gene family. The highest numbers of genes were characterized with 5 and 4 introns, which contributed about 54%, followed by 3 exons with 15% of all genes. There were only two genes, SlUBC42 and SlUBC3 without any intron.

### Gene duplication pattern analysis

The gene duplication plays a major role in diversification and evolution of altered genetic setup through adaptation to environment^18^. We identified around 47% (28 genes) of the identified E2 genes were duplicated in the tomato genome (Supplementary Table S5). The E2 genes were distributed as a single copy or more on loci. In the synteny analysis, we found fourteen single copy loci genes, seven genes with two copy loci, and Solyc03g007470.2 (SlUBC14), Solyc01g095490.2 (SlUBC5) and Solyc08g081950.2 (SlUBC41) genes with three, four and six copy loci, respectively. Surprisingly, all the duplicated genes were observed with Ka/Ks value less than 1. The non-synonymous/synonymous substitution ratio (Ka/Ks) is the robust measure of evolutionary trend of the gene. The ratio less than 1 indicates the functional constraint; equal to 1 indicates neutral selection and more than 1 implies the divergence or changes due to mutation. We have utilized orthologous E2 genes pairs from *Solanum lycopersicum* to estimate Ka, Ks and Ka/Ks ratio. Most of the genes comprised the Ka/Ks ratio less than 0.1, with the minimum value of 0.00994 for Solyc06g070980.2 (SlUBC31) gene and the maximum value of 0.4455 for Solyc12g013820.1 (SlUBC55). However, few genes with null values for Ka and Ks were also noticed. The results imply that segmental duplication is major factor for the expansion of E2 genes and clearly indicates the structural and functional conservation of E2 enzymes in tomato.

### Promoter analysis and Gene Ontology analysis

**Figure 5:**
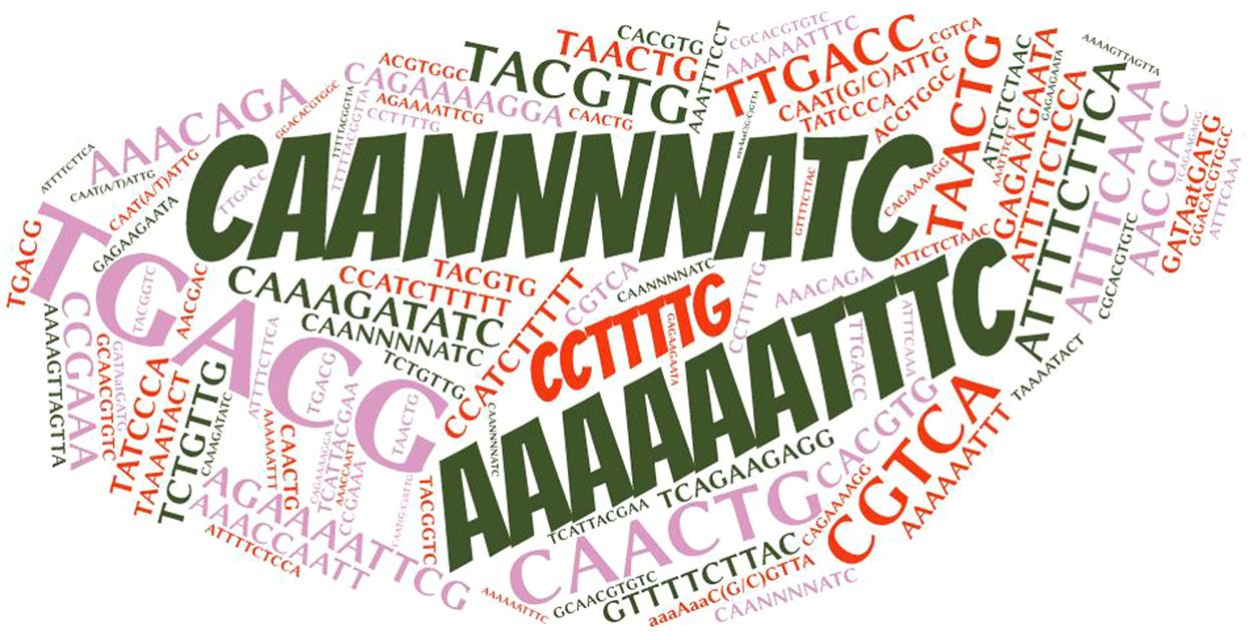
The word cloud image of promoter elements of 59 E2 ubiquitin conjugating enzymes. The size and intensity indicate frequency of the elements.

The 1000 upstream promoter analysis predicted the elements responsible for regulation of tomato E2 genes. A word cloud was generated for the promoter elements showing the frequency of the elements in the genes (Fig. 5). The occurrence of AAAAAATTTC (Heat Stress Responsive), CAANNNNATC (circadian element), TAACTG (drought inducibility), CCTTTTG (gibberellin-responsive element), ATTTTCTTCA and ATTTTCTCCA (defense and stress responsiveness), ATTTCAAA (ethylene-responsive element), GAGAAGAATA and CCATCTTTTT (salicylic acid responsiveness), TGACG and CGTCA (Jasmonic Acid Responsiveness), AACGAC (auxin-responsive element), TACGTG (abscisic acid responsiveness) elements was highest among all the gene promoters. It strongly suggests their specific role in related mechanisms. A substantial number of defence, stress responsive and hormone responsive elements were observed in the promoter sequences. It clearly indicates their probable role in biotic and hormonal pathways.

The GO analysis (Supplementary Figure S6) of the identified sequences reveals around 45% of the E2 enzymes were destined in metabolic processes (GO: 0008150), where major function is protein modification (GO: 0006464) and proteolysis (GO: 6508) including DNA metabolic processes (GO: 0006259). Few genes were also involved in catabolic processes (GO: 0009056) and nitrogen compound metabolic process (GO: 0006807). Another major part of around 34% of E2 genes is active in cell cycle mechanisms (GO: 0007049). However, 9% of the E2 genes were responsible for apoptotic process and 7.5% for stress responses (GO: 0006950).

### Gene expression analysis in different tissues

**Figure 6:**
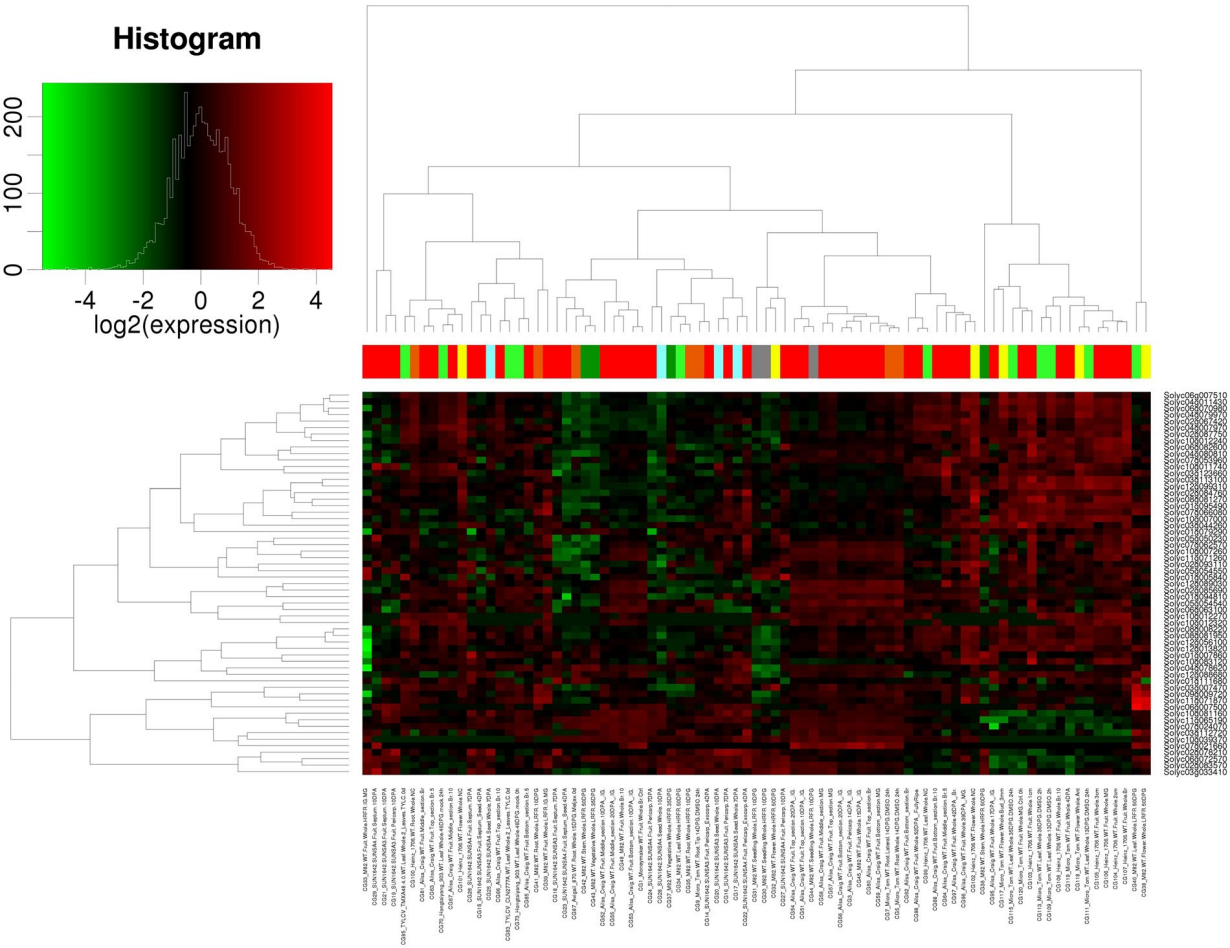
A gene expression profile of 59 tomato E2 enzyme sequences in seed, seedling, root, vegetative, leaf, flower and fruit tissue of Micro Tom, Heinz 1706, M82, Moneymaker, SUN1642, and Alisa Craig cultivars of tomato is illustrated. The normalized gene expression level is represented by a color scale histogram.

The differential expression of E2 ubiquitin-conjugating enzyme genes was analyzed in different tissues of nine cultivars of tomato (Fig. 6). The RNA sequencing data from Tom Express database^32^ was used for the gene expression analysis. The 83 different conditions were used for the analysis in major parts of tomato plant (seed, seedling, root, vegetative, leaf, flower and fruit). Most of the E2 genes were expressed in the seeds of 7DPA (seven days post-anthesis) and about half of the genes were not detected in the 10DPA (ten days post-anthesis) seeds in SUN1642 cultivar. In the seedling and vegetative stage (M82 cultivar), the expression level was relatively low with few exceptions. Heinz 1706 and MicroTom cultivars root tissues were observed with the enormous expression level of E2 genes whereas M82 and Avigail cultivars were recorded with comparatively less E2 genes expression. Heinz 1706 was marked with the highest expression of E2 genes. However, overall expression of E2 genes in flower was competitive with root tissues. Fruit septum and pericarp of SUN1642 cultivar showed expression of more than the half of the total genes in 4DPA, 7DPA and 10DPA. The SlUBC37 and SlUBC44 genes are involved in K-63 ubiquitination as found similar to UBC35 and 36 of Arabidopsis^11^, were among moderately expressed genes in the tissues. Both genes expression was not observed in seed, stem, whole vegetative and fruit septum and Alisa Craig whole fruit tissues. It was highly expressed in all tissues of Heinz cultivar compared to all other cultivars. It suggests that tomato UPS machinery also subscribes to K-63 ubiquitination in tomato tissue development and signaling. An increased expression was noticed in Heinz 1706 fruit (whole, 1 cm, 2 cm and 3 cm) and Alisa Craig compared to M82, MicroTom and Money Maker fruit. More genes were expressed in mature green stage compared to immature green stage. All E2 genes were detected in breaker stage, breaker stage +5 days and breaker stage +10 days (Whole, Top, Middle and Bottom fruit). The subsequent expression of E2 genes in top fruit part was reduced with older breaker stage fruit. Whereas, middle and bottom parts were found to be almost similar in all breaker stages, in terms of a number of genes expressed. The gene expression level is shown by Cluster 1 to 5 with highest to lowest expression (Supplementary Figure S7). It was observed that SlUBC21, 23, 29, 31, 39, 41 and 56 were highly expressed compared to other identified genes. Whereas, SlUBC3, 34, 47, 49 and 56 were not detected in most tissues or low expression was found. It is concluded that the most of the E2 genes were highly expressed in root, leave and flower tissues than other tissues. Few genes were predominantly expressed in almost all tissues. It suggests that E2 genes are involved in tomato plant growth and development in tissue specific manner. The highly expressed E2 genes are predicted to interfere the cellular signaling in tomato tissues. This expression profile provides a great insight into stage-specific activity of E2 enzymes in tomato tissues. The differential expression in different tissues of tomato in variable conditions is preliminary evidence of involvement of E2 enzymes in plant development.

### Gene expression analysis during hormone treatment and biotic stress

**Figure 7:**
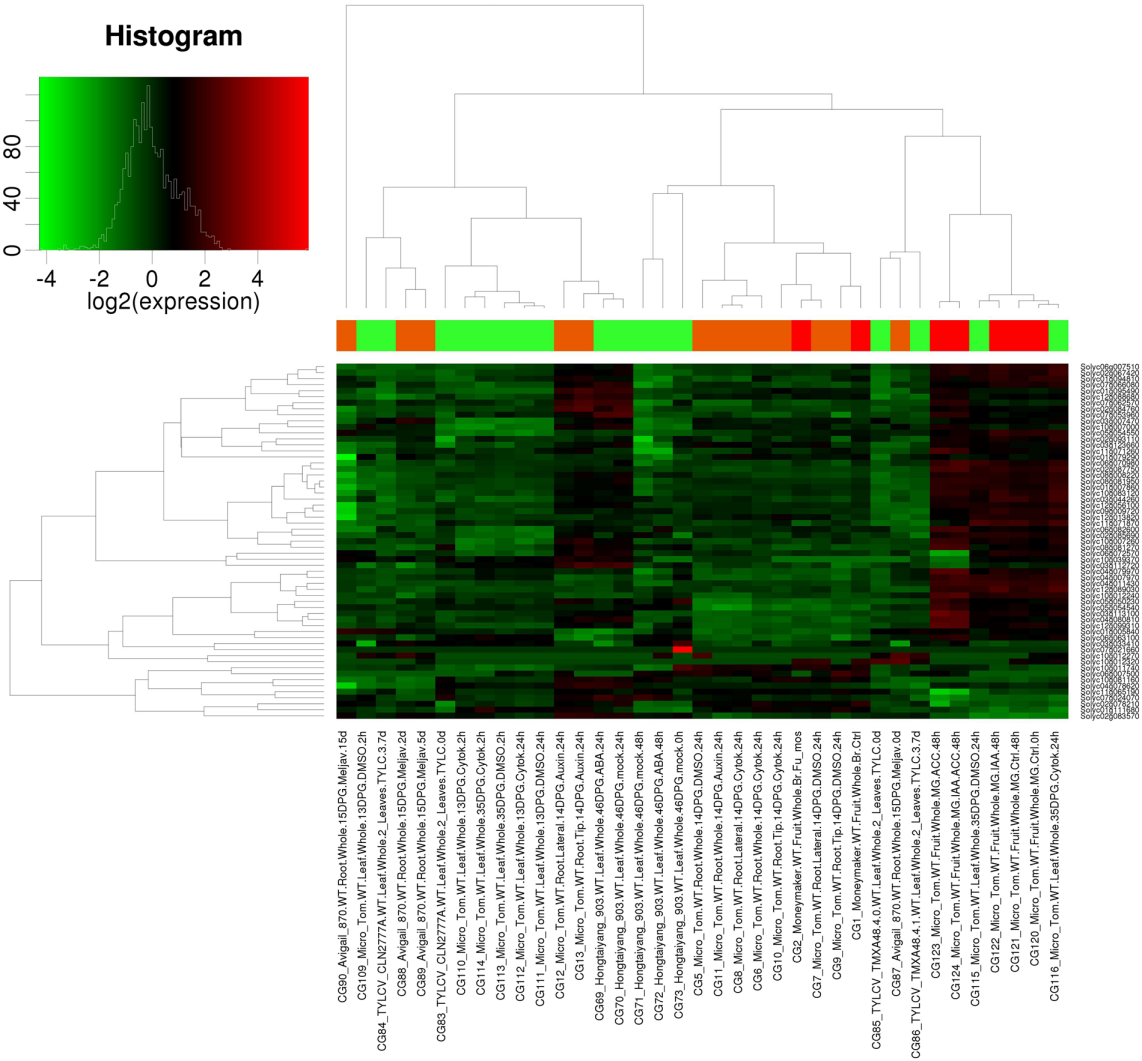
A gene expression profile of 59 tomato E2 enzyme sequences during hormone treatment and pathogen infection in root, leaf and fruit tissues of Micro Tom, Avigail, Hongtaiyamg, Moneymaker cultivars of tomato. The normalized gene expression level is represented by a color scale histogram.

The gene expression of identified E2 genes was also analyzed in hormone-treated tissues and pathogen-infected tissues (Fig. 7). The RNA sequencing pipeline of Tom Express database^32^ was used for gene expression analysis during hormone treatment and biotic stress in tomato. The E2 genes were highly expressed in root tip and lateral root tissues treated with auxin of 14 DPA in MicroTom cultivar. Leaves of MicroTom and Hongtaiyang cultivars with 13, 35 and 46 DPG stage treated with ABA, DMSO, and cytokinin showed that E2 genes were highly expressed in 35 and 46 DPG treated with DMSO, cytokinin and ABA for 24-hour incubation. Mature Green Fruit tissues of Micro Tom cultivar treated with indole acetic acid and 1-aminocyclopropane-1-arboxylic acid were observed with relatively high expression of E2 genes. The SlUBC47 and SlUBC48 genes were exceptionally highly expressed in TMXA48 and CLN2777A cultivars infected with TYLCV for 0 and 7 days. The SlUBC6, 8 and 52 E2 genes found in breaker stage of Money Maker cultivar infected with *Funneliformis mosseae*. Therefore, it can be concluded that E2 genes are selectively expressed during hormone treatment and pathogen infection. UBC37 and UBC44 genes, responsible for K-63 ubiquitination, were rarely detected in response to pathogen but these genes were expressed in response to ABA and auxin hormones. UBC44 gene expression was also detected in response to cytokinin hormone in leaf tissues where UBC37 was not expressed significantly. It indicates the presence of the K-63 ubiquitination in response to hormones and rare response under pathogen attack. In another heat map (Supplementary Figure S8), E2 enzyme genes in cluster 1, 2 and 3 are categorized from highest to lowest expression level. With these results, we can predict a major role of E2 conjugating enzymes in plant signaling during biotic stress and hormone treatment.

## Discussion

E1 ubiquitin activating, E2 ubiquitin conjugating and E3 ligase enzymes are the major part of ubiquitination process. There are only a few reports on functional significance of E2 genes in plants. We have utilized in-silico analysis for identification of E2 conjugating enzymes and obtained 59 genes with UBC domain. The physio-chemical analysis revealed hydrophilic and thermo-stable nature of the tomato E2 enzymes. The gene loss and gain over the period of time during evolution decides the genetic constituents and variability, which ultimately affect the functioning of the protein^33^. The phylogenetic analysis reveals high similarity of E2 members and gene duplication as a major factor in tomato E2 genes clan growth and enhances our understanding of diversification during evolution. The UBC superfamily domain is uniformly present in all the E2 genes and variation to this core element, as a course of evolution has resulted in an expansion. Therefore, we could identify E2 genes orthologs within all four classes with highest bootstrap values. The species-specific distribution is a possible result of evolution with conserved domains in higher plant species, and gene loss in lower eukaryotes.

Gene duplication is responsible for adaptation of genes functions as per environmental conditions and therefore, expansion of gene family accordingly. Changes in the coding region lead to either exon-intron structural change or amino acids alteration in the sequences, generating functional diversities^34^. Around 47% of the identified 59 genes were obtained with duplications from one to six copy loci, with highest events being in one copy loci. We infer here that E2 enzymes, over the time, have resisted to major functional changes. The analysis suggests that segmental gene duplication as a major determinant of expansion of E2 genes. We could observe both inter-chromosomal and intra-chromosomal segmental duplications. The values of Ka/Ks ratio were recorded <1 for all the genes where most of the genes were even less than 0.1, which strongly indicate the structural and functional conservation of the genes. We could not identify significant tandem duplications in the duplicated genes. The E2 enzymes are not significantly diversified in their native function, which is ubiquitin conjugating activity, in the tomato plant system, but their distribution may qualify them to participate and influence other cellular mechanisms. Furthermore, the expansion of gene family members is predicted due to genome instability followed by mosaic of duplication in tomato. E2 genes are conserved on synteny blocks as a result of segmental duplication.

It is well understood that the gene organization on the genome is also responsible for their diversity among species^34^. Although a large variation in the distribution of introns was observed but majority comprises similar structural organization containing 4 to 6 introns, compared to E2 genes in maize with 3–4^10^ and 5 introns in *Carica papaya* ^35^. The structural insights of E2 genes reveal strong structural integrity. Exon shuffling could be a decisive factor for the procreation of new genes in the E2 gene family. We, therefore, infer that the origin of the family members of the E2 enzymes in tomato might have undergone genetic alteration to serve the specific function of the system. However, we failed to point any significant distant functional paralog. A set of ten conserved domains was discovered in identified E2 members. The motif discovery is required to find patterns in nucleotide or protein sequences to understand the structure and function of the molecules in the system^36^. The presence of conserved domains suggests functional identity among tomato E2 candidates and also with human and Arabidopsis. The highly conserved motifs structures were preserved by evolution and predicted to participate in ubiquitination mechanism. The specific motifs in E2 genes may influence participation into particular biological function in tomato. The motif 5, 8, 9 and 10 were rarely found in human E2 members which predicts the plant specific function of these motifs. The motif 9 was not found in Arabidopsis E2 members but could be observed in tomato E2 members, which suggests its organism specific biological function. These differences are vital evidences of evolution and diversity of E2 members. Further, our data shows that tomato E2 members have composite motifs organization than Arabidopsis and human which makes the tomato an interesting candidate for the study as it may pursue a highly regulated machinery for E2 enzymes to function.

Further, the promoter analysis revealed the presence of specific elements. It can be hypothesized that these elements regulate the expression of the respective E2 genes during specific pathways. It may accomplish an imperative regulatory association with genes coding E2 conjugating enzymes to selectively express and target during biotic stress and hormone treatment. The E2 enzyme modulating abiotic stress has already been reported in the previous studies^24, 25, 37^. However, recent finding provided the role of E2 enzymes in plant immunity^38, 39^ and hormone regulation^40^ but not adequately evidenced yet and remains largely unknown. According to GO data, E2 enzymes are highly involved in proteolysis, protein modifications including stress and cell death. It adds to the predicted targets of E2 enzymes with a considerable association in many developmental processes. To get insights of the gene expression of E2 enzymes in cellular parts of the tomato plant, we utilized latest RNA sequencing data pipeline on TomExpress database. With the differential expression in root, leaf, flower and fruit, it can be implied that E2 genes are largely distributed and expressed in the tomato plant and may regulate the plant biological processes. The expression level of E2 enzymes in root, shoot, and fruits was surprisingly high. Recent reports identified that tomato E2 enzymes have a role in fruit and root development and are integrated to subsequent additional regulatory mechanisms^40, 41^. Our result supports this research as most of the E2 genes were detected in high amount during fruit and root development and predicts important regulatory mechanism during fruit development. The ubiquitin-mediated proteasomal degradation is a critical mechanism and widely affects that plant system including abiotic stress, and plant immunity^37^. It is not clear whether E2 directly influences its impact on plant growth and responses. Since the E2 enzyme is a mediator for the ubiquitination mechanism, it is reasonable to infer that additional occurrence of the E2 enzyme will aid the E3 ligase mechanism and thereby regulate the final pathway. Moreover, K-63 ubiquitination has also been studied in plants^11^ but exact biological role is not clear. We found that SlUBC37 and SlUBC44 genes are involved in K-63 ubiquitination in most of the tomato tissues and under hormone treatment but no significant detection under biotic stress. The analysis suggests that K-63 ubiquitination is also a choice of ubiquitination in many tissues, especially in root and may regulate their development. The direct interaction of E2 enzymes with its targets other than UPS is another interesting aspect of the investigation. Therefore, we can argue that the expression profile of E2 genes among various tissues can render proof of sundry roles in different plant signaling mechanisms. It also suggests that stage-specific tissues with higher expression of E2 genes, could be entailed in active ubiquitination, vital for plant development, and identification of the specific targets for regulated E3 ligases will further strengthen our understanding of protein quality control in plant signaling pathways as well as its control over plant development.

Our study of tomato E2 gene expression under biotic stress and hormone treatment provide preliminary evidence of the active involvement in signaling mechanisms. A clear picture of induction in E2 genes expression can be found in hormone treated roots, leaf and fruit tissues. An increased level of expression was also found in tomato plant infected with *Funneliformis mosseae* and *Maloidogyne javanica*. Our hypothesis is evidenced by elevated gene expression and occupancy of immune (ATTTTCTTCA and ATTTTCTCCA) and hormone (AACGAC and TACGTG) responsive element at the upstream region of E2 conjugating enzyme genes. These findings straight forward rings the bell that E2 enzymes may play the immune and hormone signaling in tomato.

In the previous studies of E2 genes, 3–4 introns were majorly found in banana, maize and papaya than 4–5 introns in tomato, suggesting additional structural and functional features in tomato E2 genes. The segmental duplication was dominant in tomato E2 genes than in banana, maize and rice. The class I motifs were the majority among banana^14^, maize^10^, papaya^35^ and tomato which serve as a primary catalytic domain for ubiquitination activity. Arabidopsis UBC 1 and 2 orthologs in tomato SlUBC31 and SlUBC12 were expressed in roots and leaves as reported in Arabidopsis, but in tomato highly expressed in fruits as well, indicating a major role in fruit and root development. The SlUBC52, ortholog of AtUBC20 along with SlUBC45 and 48 were highly expressed under biotic stress. Taken all together we conclude that tomato E2 genes are influencing biotic stress and hormone regulation. It is first time reported in the tomato that E2 enzymes have control over regulation of biotic stress and hormone signaling in tomato. The present analysis not only identifies but explores crucial aspects of E2 enzymes in tomato with a strong *in*-*silico* analysis and evidences.

In conclusion, the distribution of the E2 gene family members across the twelve chromosomes and presence of conserved motifs in almost all genes with composite elements was found in tomato. The most of the genes shared a pattern of intron-exon distribution and segmental duplication being the major influencing factor for expansion and close evolutionary relationship of the gene family. We hypothesize that E2 enzymes are showing divergence in their functions, as these are conserved and persist numerous participation in plant biological mechanisms, especially, in biotic stress and hormone exposure as evidenced by gene expression analysis. This study provided a comprehensive understanding of the distribution, evolution, and functions of the E2 ubiquitin conjugating enzyme in tomato and will create a base paradigm for understanding the functional role of E2 enzymes and ubiquitination in *Solanum lycopersicum* and other plant systems.

## Material and Methods

### Identification and bioinformatics analysis of candidate genes

To identify potential members of E2 conjugating enzymes in tomato, we used HMMER programme downloaded from HMMER (http://hmmer.org/)^29^. The UBC domains (PF00179) for E2 ubiquitin conjugating enzymes were downloaded from Pfam database (http://pfam.xfam.org/)^28^. The HMM profile was build using HMMER 3.0 package, and HMM search was performed against tomato genome with default parameters, *Solanum lycopersicum* as reference database and significane e-value of 0.01. The presence of E2 ubiquitin conjugating domain was confirmed using SMART^42^, InterPro^43^ and Pfam with default parameters. Sub-cellular localization was predicted using TargetP 1.1 server (http://www.cbs.dtu.dk/services/TargetP/)^44^. The Molecular weight, number of nucleotides, number of amino acids, GRAVY (Grand average of hydropathicity), Aliphatic index, instability index, and PI were calculated by ExPasy-Compute pI/Mw (http://web.expasy.org/compute_pi/) and Protparam (http://web.expasy.org/protparam/)^45^. The chromosomal location, intron-exon count and sequence ids were retrieved from phytozome (https://phytozome.jgi.doe.gov/pz/portal.html#)^46^, PANTHER (http://www.pantherdb.org/)^47^ and Sol Genomic Network (https://solgenomics.net/)^48^. The Gene Ontology data was retrieved from PANTHER database.

### Phylogenetic Analysis and Gene Duplication

To study evolutionary relationship of the *Solanum lycopersicum* E2 conjugating enzyme, multiple sequence alignment was performed using Clustal W using default paprameters^49^. The phylogenetic tree was constructed using full length sequences by Neighbour Joining method (N-J method) with 1000 bootstrap replications, p-distance and pairwise deletion, implemented in MEGA 7.0 version software downloaded from MEGA (http://www.megasoftware.net/)^50^. The tree was visualized by using iTOL v3 (www.itol.embl.de) online tool^51^. We have utilized the Plant Genomic Duplication Database for the analysis of gene synteny and non-synonymous/synonymous substitution ratio^52^. The tool uses CLUSTALW alignment applied in PAML package for Ks calculation and MCscan for synteny search.

### Motif analysis and Gene structure analysis

The unknown conserved motifs were identified using MEME suite, a motif-based sequence analysis tool (http://meme-suite.org/)^53^. A limit of 10 number of motifs and maximum width of 200 amino acids (number of characters in the sequence pattern), were used along with other default parameters. The Arabidopsis and human E2 ubiquitin conjugating enzyme protein sequences were obtained from previous research^5, 11^. MAST tool of MEME suite was used to compare discovered tomato motifs in Arabidopsis and human^54^. The enrichment of the discovered motifs in Arabidopsis was analyzed using AME tool^55^. An online tool Gene Structure Display System (http://gsds.cbi.pku.edu.cn/index.php)^56^ was used to characterize the structure of identified E2 ubiquitin conjugating enzymes. The length of introns and CDS were visualized using this tool.

### Chromosomal Localisation and promoter analysis

All the information related to position of each gene was retrieved from Sol genomics database. All identified genes were mapped on tomato chromosomes using ArkMap software (http://www.thearkdb.org/arkdb/). The 12 chromosomes of tomato genome were used from Ensembl plant database (http://plants.ensembl.org/index.html).

The 1000 bp promoter sequences of all identified E2 ubiquitin enzymes were fetched from the phytozome database. PlantCARE database (http://bioinformatics.psb.ugent.be/webtools/plantcare/html/) was used for identification of elements in the promoter^57^.

### Tissue specific expression

The expression of E2 ubiquitin conjugating enzyme in different tissues of the tomato was investigated using RNA sequencing data pipeline of TomExpress database (http://gbf.toulouse.inra.fr/tomexpress)^32^. The expression was analyzed in fruit, seed, seedling, root leaves, flower and vegetative tissues in different cultivars of tomato (Micro-Tom, Heinz 1706, M82, SUN1642, Hongtaiyang, Avigail and MoneyMaker). The expression data was visualized using Heat Maps and graphs generated by TomExpress database.

### Expression study under hormone treatment and biotic interaction

To investigate the expression of the identified E2 ubiquitin conjugating enzymes in biotic stress and hormone treatment conditions for vegetative tissues and fruits, Tomexpress database was used (http://gbf.toulouse.inra.fr/tomexpress)^32^. The analysis included wild type tomato cultivar (Micro-Tom, TMXA48, CLN2777A, Avigail and MoneyMaker) expression analysis. The expression data was visualized using Heat Maps and graphs generated by TomExpress database.

### Data Availability

All data generated or analysed during this study are included in this published article (and its Supplementary Information files).

## Electronic supplementary material

Supplementary Information

## References

[1] Smalle J, Vierstra RD (2004). The ubiquitin 26S proteasome proteolytic pathway. Annu. Rev. Plant Biol..

[2] Schulman BA, Harper JW (2009). Ubiquitin-like protein activation by E1 enzymes: the apex for downstream signalling pathways. Nature Reviews Molecular Cell Biology.

[3] Moon J, Parry G, Estelle M (2004). The ubiquitin-proteasome pathway and plant development. The Plant Cell.

[4] Komander D, Rape M (2012). The ubiquitin code. Annual review of biochemistry.

[5] van Wijk SJ, Timmers HM (2010). The family of ubiquitin-conjugating enzymes (E2s): deciding between life and death of proteins. The FASEB Journal.

[6] Özkan E, Yu H, Deisenhofer J (2005). Mechanistic insight into the allosteric activation of a ubiquitin-conjugating enzyme by RING-type ubiquitin ligases. Proceedings of the National Academy of Sciences of the United States of America.

[7] Eddins MJ, Carlile CM, Gomez KM, Pickart CM, Wolberger C (2006). Mms2–Ubc13 covalently bound to ubiquitin reveals the structural basis of linkage-specific polyubiquitin chain formation. Nature structural & molecular biology.

[8] Ye Y, Rape M (2009). Building ubiquitin chains: E2 enzymes at work. Nature reviews Molecular cell biology.

[9] Bae H, Kim WT (2014). Classification and interaction modes of 40 rice E2 ubiquitin-conjugating enzymes with 17 rice ARM-U-box E3 ubiquitin ligases. Biochemical and biophysical research communications.

[10] Jue D (2015). Genome-wide identification, phylogenetic and expression analyses of the ubiquitin-conjugating enzyme gene family in maize. PloS one.

[11] Kraft E (2005). Genome Analysis and Functional Characterization of the E2 and RING-Type E3 Ligase Ubiquitination Enzymes of Arabidopsis. Plant Physiology.

[12] Jones D, Crowe E, Stevens TA, Candido EPM (2001). Functional and phylogenetic analysis of the ubiquitylation system in Caenorhabditis elegans: ubiquitin-conjugating enzymes, ubiquitin-activating enzymes, and ubiquitin-like proteins. Genome biology.

[13] Michelle C, Vourc’h P, Mignon L, Andres CR (2009). What was the set of ubiquitin and ubiquitin-like conjugating enzymes in the eukaryote common ancestor?. Journal of molecular evolution.

[14] Dong C (2016). The banana E2 gene family: Genomic identification, characterization, expression profiling analysis. Plant Science.

[15] Semple CA, Group RG (2003). The comparative proteomics of ubiquitination in mouse. Genome research.

[16] E. Z, Zhang Y, Li T, Wang L, Zhao H (2015). Characterization of the Ubiquitin-Conjugating Enzyme Gene Family in Rice and Evaluation of Expression Profiles under Abiotic Stresses and Hormone Treatments. PLoS ONE.

[17] Yang S (2008). Repetitive element-mediated recombination as a mechanism for new gene origination in Drosophila. PLoS Genet.

[18] Taylor JS, Raes J (2004). Duplication and divergence: the evolution of new genes and old ideas. Annu. Rev. Genet..

[19] Shikata, M. & Ezura, H. Micro-Tom Tomato as an Alternative Plant Model System: Mutant Collection and Efficient Transformation. *Plant Signal Transduction*: *Methods and Protocols*, 47–55 (2016). 10.1007/978-1-4939-3115-6_5 26577780

[20] Arie T, Takahashi H, Kodama M, Teraoka T (2007). Tomato as a model plant for plant-pathogen interactions. Plant Biotechnology.

[21] Lyzenga, W. J. & Stone, S. L. Abiotic stress tolerance mediated by protein ubiquitination. *Journal of experimental botany*, err310 (2011). 10.1093/jxb/err310 22016431

[22] Jeon EH (2012). Ectopic expression of ubiquitin-conjugating enzyme gene from wild rice, OgUBC1, confers resistance against UV-B radiation and Botrytis infection in Arabidopsis thaliana. Biochemical and biophysical research communications.

[23] Hamera S, Mural RM, Liu Y, Zeng L (2014). The tomato ubiquitin-conjugating enzyme variant Suv, but not SlUev1C and SlUev1D regulates Fen-mediated programmed cell death in Nicotiana benthamiana. Plant signaling & behavior.

[24] Chung E (2013). Overexpression of VrUBC1, a mung bean E2 ubiquitin-conjugating enzyme, enhances osmotic stress tolerance in Arabidopsis. PloS one.

[25] Zhou G-A, Chang R-Z, Qiu L-J (2010). Overexpression of soybean ubiquitin-conjugating enzyme gene GmUBC2 confers enhanced drought and salt tolerance through modulating abiotic stress-responsive gene expression in Arabidopsis. Plant molecular biology.

[26] Wan X, Mo A, Liu S, Yang L, Li L (2011). Constitutive expression of a peanut ubiquitin-conjugating enzyme gene in Arabidopsis confers improved water-stress tolerance through regulation of stress-responsive gene expression. Journal of bioscience and bioengineering.

[27] Cui F (2012). Arabidopsis ubiquitin conjugase UBC32 is an ERAD component that functions in brassinosteroid-mediated salt stress tolerance. The Plant Cell.

[28] Finn RD (2016). The Pfam protein families database: towards a more sustainable future. Nucleic acids research.

[29] Eddy SR (1998). Profile hidden Markov models. Bioinformatics.

[30] Papaleo E (2012). Loop 7 of E2 enzymes: an ancestral conserved functional motif involved in the E2-mediated steps of the ubiquitination cascade. PloS one.

[31] Schumacher F-R, Wilson G, Day CL (2013). The N-terminal extension of UBE2E ubiquitin-conjugating enzymes limits chain assembly. Journal of molecular biology.

[32] Maza E, Frasse P, Senin P, Bouzayen M, Zouine M (2013). Comparison of normalization methods for differential gene expression analysis in RNA-Seq experiments: A matter of relative size of studied transcriptomes. Communicative & integrative biology.

[33] Albalat, R. & Canestro, C. Evolution by gene loss. *Nat Rev Genet* **17**, 379–391, doi:10.1038/nrg.2016.39http://www.nature.com/nrg/journal/v17/n7/abs/nrg.2016.39.html#supplementary-information (2016). 10.1038/nrg.2016.39 27087500

[34] Xu G, Guo C, Shan H, Kong H (2012). Divergence of duplicate genes in exon–intron structure. Proceedings of the National Academy of Sciences.

[35] Jue D (2017). Characterization and expression analysis of genes encoding ubiquitin conjugating domain-containing enzymes in Carica papaya. PloS one.

[36] Žiarovská J, Záhorský M, Gálová Z, Hricová A (2015). Bioinformatic approach in the identification of Arabidopsis gene homologous in Amaranthus. Potravinarstvo.

[37] Sharma B, Joshi D, Yadav PK, Gupta AK, Bhatt TK (2016). Role of ubiquitin-mediated degradation system in plant biology. Frontiers in Plant Science.

[38] Zhou B (2017). A Subset of Ubiquitin-Conjugating Enzymes Is Essential for Plant Immunity. Plant Physiology.

[39] Mural RV (2013). The Tomato Fni3 Lysine-63–Specific Ubiquitin-Conjugating Enzyme and Suv Ubiquitin E2 Variant Positively Regulate Plant Immunity. The Plant Cell.

[40] Wen R (2014). UBC13, an E2 enzyme for Lys63‐linked ubiquitination, functions in root development by affecting auxin signaling and Aux/IAA protein stability. The Plant Journal.

[41] Wang Y (2014). Tomato nuclear proteome reveals the involvement of specific E2 ubiquitin-conjugating enzymes in fruit ripening. Genome biology.

[42] Schultz J, Milpetz F, Bork P, Ponting CP (1998). SMART, a simple modular architecture research tool: identification of signaling domains. Proceedings of the National Academy of Sciences.

[43] Mitchell, A. *et al*. The InterPro protein families database: the classification resource after 15 years. *Nucleic acids research*, gku1243 (2014). 10.1093/nar/gku1243 PMC4383996 25428371

[44] Emanuelsson O, Nielsen H, Brunak S, Von Heijne G (2000). Predicting subcellular localization of proteins based on their N-terminal amino acid sequence. Journal of molecular biology.

[45] Gasteiger, E. *et al*. *Protein identification and analysis tools on the ExPASy server* (Springer, 2005). 10.1385/1-59259-584-7:531 10027275

[46] Goodstein DM (2012). Phytozome: a comparative platform for green plant genomics. Nucleic acids research.

[47] Thomas PD (2003). PANTHER: a library of protein families and subfamilies indexed by function. Genome research.

[48] Fernandez-Pozo N (2015). The Sol Genomics Network (SGN)—from genotype to phenotype to breeding. Nucleic acids research.

[49] Larkin MA (2007). Clustal W and Clustal X version 2.0. bioinformatics.

[50] Kumar, S., Stecher, G. & Tamura, K. MEGA7: Molecular Evolutionary Genetics Analysis version 7.0 for bigger datasets. *Molecular biology and evolution*, msw054 (2016). 10.1093/molbev/msw054 PMC8210823 27004904

[51] Letunic I, Bork P (2007). Interactive Tree Of Life (iTOL): an online tool for phylogenetic tree display and annotation. Bioinformatics.

[52] Lee T-H, Tang H, Wang X, Paterson AH (2013). PGDD: a database of gene and genome duplication in plants. Nucleic acids research.

[53] Bailey, T. L. & Elkan, C. Fitting a mixture model by expectation maximization to discover motifs in bipolymers (1994). 7584402

[54] Bailey TL, Gribskov M (1998). Combining evidence using p-values: application to sequence homology searches. Bioinformatics (Oxford, England).

[55] McLeay RC, Bailey TL (2010). Motif Enrichment Analysis: a unified framework and an evaluation on ChIP data. BMC bioinformatics.

[56] Hu, B. *et al*. GSDS 2.0: an upgraded gene feature visualization server. *Bioinformatics*, btu817 (2014). 10.1093/bioinformatics/btu817 PMC4393523 25504850

[57] Rombauts S, Déhais P, Van Montagu M, Rouzé P (1999). PlantCARE, a plant cis-acting regulatory element database. Nucleic acids research.

