# Author Correction: Genome-wide identification and expression analysis of E2 ubiquitin-conjugating enzymes in tomato

**DOI:** 10.1038/s41598-018-24611-9

**Published:** 2018-04-25

**Authors:** Bhaskar Sharma, Tarun Kumar Bhatt

**Affiliations:** Department of Biotechnology, Central University of Rajasthan, Bandarsindri, Ajmer 305817 India

Correction to: *Scientific Reports* 10.1038/s41598-017-09121-4, published online 17 August 2017

The Acknowledgements section in this Article is incomplete.

“The author’s laboratory is supported by DST grant of Govt. of India.”

should read:

“Authors would like to thank Prof. A. K. Gupta for his support and valuable inputs. The author’s laboratory is supported by DST grant of Govt. of India.”

