# Supplementary material for: Genome-wide identification and expression analysis of E2 ubiquitin-conjugating enzymes in tomato
Source: Sci Rep. 2017 Aug 17;7:8613. doi: 10.1038/s41598-017-09121-4 (PMC5561181; doi:10.1038/s41598-017-09121-4)
Supplement: Supplementary file 1 — Supplementary Information [file 41598_2017_9121_MOESM1_ESM.docx]

Genome-wide identification and expression analysis of E2 ubiquitin conjugating enzymes in tomato

Bhaskar Sharma ^1^ and Tarun Kumar Bhatt^1*^

^1^ Department of Biotechnology, Central University of Rajasthan, Bandarsindri, Ajmer, India 305817

*Correspondence: [tarun@curaj.ac.in](mailto:tarun@curaj.ac.in)

**Supplementary Information**

**Supplementary Table S1**

**Supplementary Figure S2**

**Supplementary Table S3**

**Supplementary Figure S4**

**Supplementary Table S5**

**Supplementary Figure S6**

**Supplementary Figure S7**

**Supplementary Figure S8**

| E2 Conjugating Enzyme | Gene Name | Class | pI | Mol. Wt. | Amino Acids | Intron | Subcellular Localization | Instability Index | Aliphatic Index | Grand Average of Hydropathicity (GRAVY) | Gene Size | Chromosomal Position |
| --- | --- | --- | --- | --- | --- | --- | --- | --- | --- | --- | --- | --- |
| Solyc04g078620.2 | UBC22 | 1 | 6.95 | 18031.43 | 160 | 4 | Other | 53.05 | 60.31 | -0.711 | 4119 | SL2.40ch04:60902088-60906206 |
| Solyc08g081950.2 | UBC41 | 1 | 7.8 | 13492.62 | 119 | 2 | Other | 44.29 | 77.9 | -0.248 | 1423 | SL2.40ch08:62024412-62025834 |
| Solyc06g007500.1 | UBC28 | 4 | 5.14 | 30363.35 | 262 | 1 | Other | 51.55 | 86.68 | -0.55 | 2961 | SL2.40ch06:1495779-1498739 |
| Solyc10g081160.1 | UBC50 | 3 | 8.97 | 28511.78 | 263 | 5 | Other | 42.08 | 87.19 | -0.359 | 2129 | SL2.40ch10:61623908-61626036 |
| Solyc11g071260.1 | UBC53 | 1 | 8.69 | 17731.45 | 157 | 4 | Other | 49.06 | 78.34 | -0.328 | 4524 | SL2.40ch11:51878146-51882669 |
| Solyc03g044260.2 | UBC16 | 1 | 8.33 | 17937.52 | 160 | 4 | Mitochondria | 54.18 | 73.12 | -0.483 | 5159 | SL2.40ch03:12566720-12571878 |
| Solyc01g007860.2 | UBC2 | 2 | 6.2 | 16544.81 | 146 | 3 | Other | 29.72 | 69.32 | -0.583 | 4188 | SL2.40ch01:2018307-2022494 |
| Solyc08g081270.2 | UBC40 | 3 | 4.47 | 20853.53 | 183 | 5 | Other | 53.42 | 65.46 | -0.614 | 6149 | SL2.40ch08:61492227-61498375 |
| Solyc07g066080.2 | UBC38 | 1 | 8.42 | 16435.91 | 148 | 3 | Other | 48.43 | 69.86 | -0.382 | 1922 | SL2.40ch07:64830179-64832100 |
| Solyc02g085690.2 | UBC11 | 4 | 8.79 | 25704.71 | 226 | 6 | Secretory Pathway | 43.8 | 84.07 | -0.345 | 3898 | SL2.40ch02:43086573-43090470 |
| Solyc01g094810.2 | UBC4 | 3 | 4.44 | 20780.44 | 184 | 6 | Other | 53.61 | 70.49 | -0.423 | 3318 | SL2.40ch01:77982018-77985335 |
| Solyc02g093110.2 | UBC13 | 1 | 7.71 | 18066.69 | 160 | 4 | Other | 52.68 | 72.5 | -0.51 | 3983 | SL2.40ch02:48605264-48609246 |
| Solyc10g039370.1 | UBC49 | 1 | 5.25 | 9001.33 | 80 | 3 | Other | 41.28 | 63.25 | -0.28 | 2213 | SL2.40ch10:21356872-21359084 |
| Solyc03g033410.2 | UBC15 | 1 | 8.45 | 16698.2 | 148 | 3 | Mitochondria | 50.47 | 81.08 | -0.344 | 2032 | SL2.40ch03:9256675-9258706 |
| Solyc04g079970.2 | UBC23 | 4 | 8.29 | 21014.12 | 183 | 4 | Other | 52.87 | 72.95 | -0.566 | 3830 | SL2.40ch04:61876931-61880760 |
| Solyc02g067420.2 | UBC7 | 1 | 5.63 | 17292.48 | 152 | 5 | Other | 69.07 | 70 | -0.575 | 4365 | SL2.40ch02:32182792-32187156 |
| Solyc10g083120.1 | UBC51 | 2 | 6.2 | 16602.87 | 146 | 3 | Other | 30.24 | 71.3 | -0.575 | 4511 | SL2.40ch10:62310434-62314944 |
| Solyc01g111680.2 | UBC6 | 4 | 4.65 | 109976.14 | 992 | 7 | Other | 38.19 | 80.13 | -0.443 | 6894 | SL2.40ch01:89632344-89639237 |
| Solyc10g012320.1 | UBC48 | 3 | 5.83 | 15649.22 | 134 | 2 | Secretory Pathway | 26.91 | 98.13 | 0.057 | 596 | SL2.40ch10:4969596-4970191 |
| Solyc09g009720.1 | UBC42 | 1 | 5.15 | 18802.21 | 167 | 0 | Other | 45.96 | 75.87 | -0.406 | 504 | SL2.40ch09:3123571-3124074 |
| Solyc06g072570.2 | UBC32 | 3 | 4.95 | 21371.31 | 194 | 4 | Other | 33.44 | 88.51 | -0.24 | 4095 | SL2.40ch06:41145493-41149587 |
| Solyc10g007000.2 | UBC43 | 4 | 6.63 | 72834.66 | 667 | 7 | Other | 38.9 | 66.91 | -0.438 | 5126 | SL2.40ch10:1404113-1409238 |
| Solyc12g099310.1 | UBC59 | 3 | 5.17 | 34087.35 | 301 | 4 | Other | 58.32 | 70.07 | -0.686 | 3307 | SL2.40ch12:64838370-64841676 |
| Solyc06g070980.2 | UBC31 | 1 | 5.37 | 17334.54 | 152 | 5 | Mitochondria | 73.04 | 70 | -0.577 | 5584 | SL2.40ch06:39977108-39982691 |
| Solyc07g024070.1 | UBC35 | 3 | 8.35 | 71871.99 | 648 | 6 | Mitochondria | 60.55 | 74.83 | -0.608 | 3422 | SL2.40ch07:24142104-24145525 |
| Solyc06g007510.2 | UBC29 | 1 | 7.72 | 16522.09 | 148 | 4 | Other | 45.84 | 76.49 | -0.289 | 5112 | SL2.40ch06:1501286-1506397 |
| Solyc11g071870.1 | UBC54 | 3 | 4.41 | 21059.55 | 185 | 5 | Other | 45.57 | 70.49 | -0.621 | 2333 | SL2.40ch11:52295316-52297648 |
| Solyc03g123660.2 | UBC19 | 3 | 8.93 | 26924.2 | 239 | 8 | Other | 50.55 | 75.82 | -0.368 | 3948 | SL2.40ch03:64499904-64503851 |
| Solyc08g008220.2 | UBC39 | 1 | 7.71 | 16480.05 | 148 | 4 | Other | 46.51 | 76.49 | -0.291 | 3681 | SL2.40ch08:2668832-2672512 |
| Solyc06g082600.2 | UBC33 | 1 | 7.72 | 16522.09 | 148 | 5 | Other | 45.84 | 76.49 | -0.289 | 4487 | SL2.40ch06:44657000-44661486 |
| Solyc11g065190.1 | UBC52 | 2 | 5.19 | 19998.78 | 181 | 5 | Cytoplasm | 57.06 | 75.41 | -0.285 | 1960 | SL2.40ch11:47601564-47603523 |
| Solyc05g054540.2 | UBC26 | 1 | 5.06 | 18877.25 | 170 | 5 | Cytoplasm | 54.62 | 69.94 | -0.433 | 3668 | SL2.40ch05:63586295-63589962 |
| Solyc07g021660.1 | UBC34 | 1 | 5.46 | 16269.65 | 138 | 2 | Secretory Pathway | 61.75 | 77.68 | -0.24 | 1026 | SL2.40ch07:20896349-20897374 |
| Solyc10g007260.2 | UBC44 | 1 | 6.74 | 17219.87 | 153 | 6 | Other | 49.9 | 92.55 | -0.319 | 5255 | SL2.40ch10:1673007-1678261 |
| Solyc07g053960.2 | UBC36 | 4 | 4.52 | 51311.59 | 463 | 5 | Other | 48.42 | 69.44 | -0.538 | 3685 | SL2.40ch07:59681394-59685078 |
| Solyc01g095490.2 | UBC5 | 1 | 7.72 | 16546.15 | 148 | 4 | Other | 40.56 | 77.09 | -0.293 | 4704 | SL2.40ch01:78493186-78497889 |
| Solyc01g079290.1 | UBC3 | 4 | 7.66 | 33136.03 | 284 | 0 | Other | 43 | 81.34 | -0.471 | 855 | SL2.40ch01:70883001-70883855 |
| Solyc10g012270.1 | UBC47 | 1 | 5.69 | 14650 | 127 | 2 | Secretory Pathway | 22.54 | 94.33 | 0.084 | 577 | SL2.40ch10:4846617-4847193 |
| Solyc04g007970.2 | UBC20 | 2 | 5.12 | 18048.5 | 160 | 5 | Other | 34.47 | 66.88 | -0.617 | 8476 | SL2.40ch04:1622530-1631005 |
| Solyc03g112720.2 | UBC17 | 1 | 8.93 | 16622.04 | 149 | 4 | Mitochondria | 55.17 | 71.34 | -0.494 | 2855 | SL2.40ch03:57120139-57122993 |
| Solyc12g056100.1 | UBC56 | 1 | 7.72 | 16522.09 | 148 | 3 | Other | 45.77 | 75.81 | -0.279 | 4301 | SL2.40ch12:47426578-47430878 |
| Solyc05g050230.2 | UBC25 | 1 | 7.71 | 16452 | 148 | 4 | Other | 44.85 | 75.14 | -0.316 | 3668 | SL2.40ch05:59425821-59429488 |
| Solyc03g007470.2 | UBC14 | 1 | 7.72 | 16577.13 | 148 | 4 | Other | 48.2 | 75.14 | -0.349 | 3981 | SL2.40ch03:2043582-2047562 |
| Solyc12g013820.1 | UBC55 | 4 | 6.58 | 81445.63 | 742 | 7 | Cytoplasm | 46.19 | 71.21 | -0.324 | 6307 | SL2.40ch12:4603477-4609783 |
| Solyc10g012240.2 | UBC46 | 3 | 4.45 | 20848.49 | 183 | 5 | Other | 53.03 | 64.97 | -0.566 | 3946 | SL2.40ch10:4720211-4724156 |
| Solyc12g089030.1 | UBC58 | 4 | 6.08 | 20789.72 | 183 | 4 | Other | 39.98 | 83.61 | -0.401 | 2035 | SL2.40ch12:62630033-62632067 |
| Solyc04g080810.2 | UBC24 | 2 | 8.35 | 18428.1 | 161 | 5 | Mitochondria | 63.33 | 76.89 | -0.424 | 4412 | SL2.40ch04:62479013-62483424 |
| Solyc10g011740.2 | UBC45 | 1 | 7.72 | 16504.07 | 148 | 3 | Other | 46.28 | 75.81 | -0.31 | 3804 | SL2.40ch10:3983198-3987001 |
| Solyc01g005840.2 | UBC1 | 2 | 8.52 | 19393.09 | 181 | 3 | Other | 33.22 | 72.21 | -0.19 | 3004 | SL2.40ch01:571846-574849 |
| Solyc06g063100.2 | UBC30 | 3 | 7.68 | 26089.33 | 231 | 8 | Other | 51.01 | 81.82 | -0.236 | 3517 | SL2.40ch06:36248969-36252485 |
| Solyc04g011430.2 | UBC21 | 1 | 5.08 | 18646 | 165 | 5 | Other | 59.21 | 73.82 | -0.462 | 3588 | SL2.40ch04:3885139-3888726 |
| Solyc03g113100.2 | UBC18 | 1 | 5.37 | 17349.49 | 152 | 5 | Mitochondria | 73.23 | 71.91 | -0.594 | 5707 | SL2.40ch03:57431345-57437051 |
| Solyc02g084760.2 | UBC10 | 2 | 7 | 18343.85 | 161 | 5 | Cytoplasm | 70.55 | 76.89 | -0.451 | 4607 | SL2.40ch02:42439595-42444201 |
| Solyc07g062570.2 | UBC37 | 1 | 6.74 | 17233.9 | 153 | 7 | Other | 49.03 | 93.79 | -0.303 | 5540 | SL2.40ch07:62472541-62478080 |
| Solyc02g078210.2 | UBC8 | 4 | 4.82 | 102829.6 | 925 | 7 | Other | 42.68 | 78.56 | -0.335 | 5780 | SL2.40ch02:37547439-37553218 |
| Solyc12g088680.1 | UBC57 | 1 | 8.4 | 18042.46 | 160 | 4 | Other | 53.86 | 59.12 | -0.671 | 2130 | SL2.40ch12:62404569-62406698 |
| Solyc02g083570.2 | UBC9 | 1 | 8.36 | 16699.19 | 148 | 3 | Other | 49.14 | 73.18 | -0.428 | 1479 | SL2.40ch02:41507853-41509331 |
| Solyc02g087750.2 | UBC12 | 1 | 5.37 | 17341.57 | 152 | 5 | Mitochondria | 67.74 | 70 | -0.559 | 5110 | SL2.40ch02:44634950-44640059 |
| Solyc05g054550.2 | UBC27 | 2 | 5.05 | 19011.48 | 170 | 5 | Other | 56.66 | 71.65 | -0.436 | 3947 | SL2.40ch05:63593121-63597067 |

**Supplementary Table S1:** The Sol genomics ID, UBC names, class, pI, molecular weight, number of amino acids, exons, introns, sub-cellular localization, instability index, aliphatic index, GRAVY (Grand Average of Hydropathicity), number of nucleotides and chromosomal location of identified 59 E2 enzymes are given in the table.

**
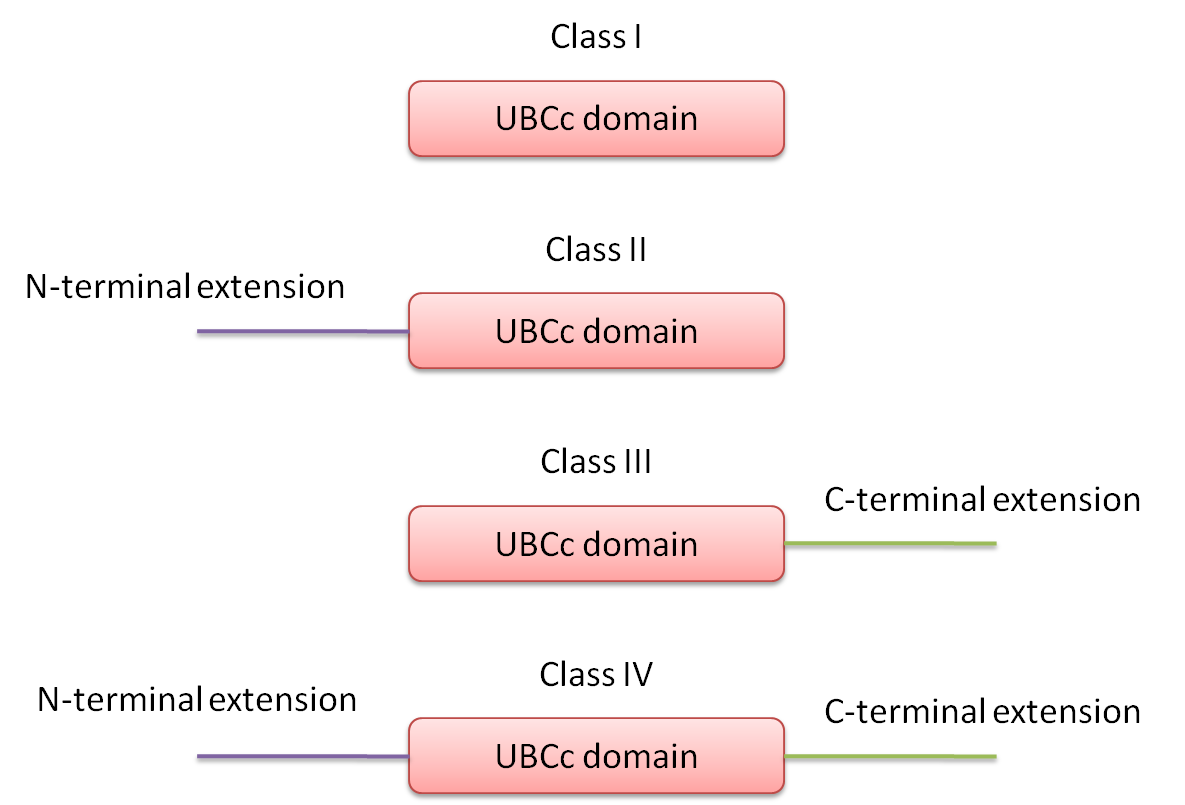
**

**Supplementary Figure S2:** The diagrammatic representation of E2 enzyme classes

| **Scanned Motifs** | | | |  |
| --- | --- | --- | --- | --- |
| Motif Number | Width | Tomato Motif Sequence | Best Possible Match |  |
| 1 | 41 | GPPDTPYEGGVFKFTIHFPEDYPFKPPKVRFRTKVFHPNIN | GPPDTPYEGGVFKFTIHFPEDYPFKPPKVRFRTKVFHPNIN |  |
| 2 | 33 | CSLLTDPNPDDPLNPEAAHMYMTDRAEYERRVR | CSLLTDPNPDDPLNPEAAHMYMTDRAEYERRVR |  |
| 3 | 29 | MARKRIQKEWKDWQKDPPTGCSAGPVDED | MARKRIQKEWKDWQKDPPTGCSAGPVDED |  |
| 4 | 11 | CLDILKEQWSP | CLDILKEQWSP |  |
| 5 | 126 | LYECGKVCLSLLNTWTGRGNEKWIPRSSTMLQVLVSIQGLILNAKPYFNEPGYDAQIGTAEGERNSLSYNENTFILNLKTMVYCMRRPPKHFEDLVRGHFRQRAQDILVACKAYMEGAPVGSLVRG | LYECGKVCLSLLNTWTGRGNEKWIPRSSTMLQVLVSIQGLILNAKPYFNEPGYDAQIGTAEGERNSLSYNENTFILNLKTMVYCMRRPPKHFEDLVRGHFRQRAQDILVACKAYMEGAPVGSLVRG |  |
| 6 | 11 | ALTISKILLSI | ALTISKILLSI |  |
| 7 | 8 | MFHWQATI | MFHWQATI |  |
| 8 | 8 | WTQKYAMG | WTQKYAMG |  |
| 9 | 57 | KVKEKQREQAENANGKPPIKKQTAGELRLHKDISELNLPRTCTISFPNGKDDLMNFE | KVKEKQREQAENANGKPPIKKQTAGELRLHKDISELNLPRTCTISFPNGKDDLMNFE |  |
| 10 | 29 | EMDLMKLMMSDYKVEMINDGMQEFYVHFH | EMDLMKLMMSDYKVEMINDGMQEFYVHFH |  |
| **Motif Enrichment Analysis** | | | |  |
| Enriched tomato motif in *Arabidopsis thaliana* | | | Enriched tomato motif in *Homo sapiens* |  |
| GPPDTPYEGGVFKFTIHFPEDYPFKPPKVRFRTKVFHPNIN | | | GPPDTPYEGGVFKFTIHFPEDYPFKPPKVRFRTKVFHPNIN |  |
| MARKRIQKEWKDWQKDPPTGCSAGPVDED | | | MARKRIQKEWKDWQKDPPTGCSAGPVDED | |
| CLDILKEQWSP | | | CSLLTDPNPDDPLNPEAAHMYMTDRAEYERRVR | |
| CSLLTDPNPDDPLNPEAAHMYMTDRAEYERRVR | | | CLDILKEQWSP | |
| ALTISKILLSI | | | ALTISKILLSI | |

**Supplementary Table S3:** The ten identified conserved motifs for 59 E2 ubiquitin conjugating enzymes and enriched motifs in Arabidopsis and human.

**
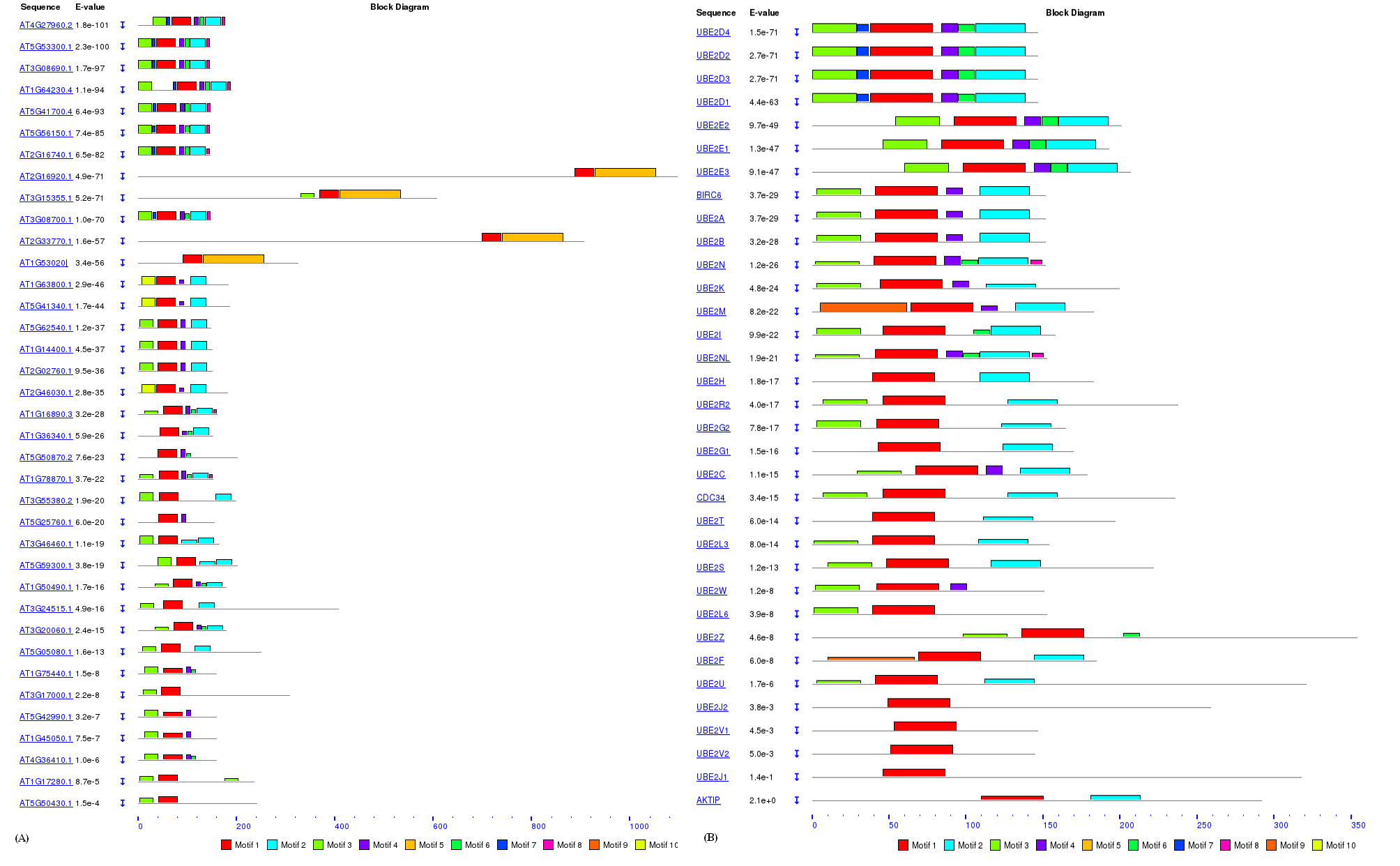
**

**Supplementary Figure S4:** The status of discovered tomato motifs in Arabidopsis and human.

| The ratio of Ka/Ks and synteny block details of tomato E2 ubiquitin conjugating enzyme retrieved from Plant Genome Duplication Database (PGDD). | | | | | | | |
| --- | --- | --- | --- | --- | --- | --- | --- |
| **Block Number** | **Block Score** | **E-value** | **Locus 1** | **Locus 2** | **Ka** | **Ks** | **Ka/Ks** |
| 225 | 3833 | 2.00E-106 | **Solyc04g078620.2** | Solyc12g088680.1 | 0.0808 | 0.8085 | 0.099938 |
| 96 | 1378 | 1.00E-92 | Solyc02g093110.2 | **Solyc04g078620.2** | 0.1049 | 1.8969 | 0.055301 |
| 337 | 1030 | 1.00E-84 | **Solyc08g081950.2** | Solyc12g056100.1 | 0.022 | 0.5932 | 0.037087 |
| 332 | 470 | 1.00E-85 | **Solyc08g081950.2** | Solyc10g011740.2 | 0 | 0 | 0 |
| 330 | 2859 | 4.00E-84 | Solyc08g008220.2 | **Solyc08g081950.2** | 0.0296 | 0.5094 | 0.058108 |
| 238 | 250 | 5.00E-84 | Solyc05g050230.2 | **Solyc08g081950.2** | 0 | 0 | 0 |
| 178 | 327 | 2.00E-86 | Solyc03g007470.2 | **Solyc08g081950.2** | 0.0183 | 1.4094 | 0.012984 |
| 44 | 1369 | 4.00E-84 | Solyc01g095490.2 | **Solyc08g081950.2** | 0 | 0 | 0 |
| 82 | 1218 | 5.00E-113 | Solyc02g093110.2 | **Solyc03g044260.2** | 0.052 | 0.5674 | 0.091646 |
| 56 | 236 | 1.00E-102 | **Solyc01g007860.2** | Solyc10g083120.1 | 0.009 | 0.4642 | 0.019388 |
| 332 | 470 | 1.00E-107 | **Solyc08g081270.2** | Solyc10g012240.2 | 0.0957 | 2.5009 | 0.038266 |
| 44 | 1369 | 2.00E-106 | Solyc01g094810.2 | **Solyc08g081270.2** | 0.0882 | 2.4893 | 0.035432 |
| 100 | 1872 | 2.00E-101 | **Solyc02g085690.2** | Solyc04g079970.2 | 0.1731 | 1.6599 | 0.104283 |
| 58 | 1751 | 1.00E-126 | **Solyc01g094810.2** | Solyc10g012240.2 | 0.0416 | 0.6088 | 0.068331 |
| 75 | 825 | 1.00E-24 | Solyc02g067420.2 | **Solyc02g093110.2** | 0 | 0 | 0 |
| 81 | 2116 | 4.00E-103 | Solyc02g083570.2 | **Solyc03g033410.2** | 0.0397 | 0.5479 | 0.072458 |
| 225 | 3833 | 2.00E-128 | **Solyc04g079970.2** | Solyc12g089030.1 | 0.0484 | 0.616 | 0.078571 |
| 112 | 555 | 2.00E-106 | **Solyc02g067420.2** | Solyc06g070980.2 | 0.0523 | 3.7189 | 0.014063 |
| 78 | 2681 | 5.00E-108 | **Solyc02g067420.2** | Solyc02g087750.2 | 0.0286 | 0.8014 | 0.035688 |
| 373 | 565 | 0 | **Solyc10g007000.2** | Solyc12g013820.1 | 0.3577 | 0.8789 | 0.406986 |
| 311 | 266 | 3.00E-127 | Solyc07g053960.2 | **Solyc10g007000.2** | 0.4641 | 2.1141 | 0.219526 |
| 151 | 1073 | 1.00E-110 | Solyc03g113100.2 | **Solyc06g070980.2** | 0.0142 | 0.6033 | 0.023537 |
| 113 | 440 | 8.00E-112 | Solyc02g087750.2 | **Solyc06g070980.2** | 0.02 | 2.1274 | 0.009401 |
| 254 | 1719 | 3.00E-110 | **Solyc06g007510.2** | Solyc06g082600.2 | 0.0029 | 0.593 | 0.00489 |
| 150 | 1328 | 6.00E-137 | **Solyc03g123660.2** | Solyc06g063100.2 | 0.0718 | 0.5653 | 0.127012 |
| 338 | 422 | 5.00E-107 | **Solyc08g008220.2** | Solyc12g056100.1 | 0.0177 | 0.6293 | 0.028126 |
| 46 | 509 | 4.00E-105 | Solyc01g095490.2 | **Solyc08g008220.2** | 0 | 0 | 0 |
| 209 | 701 | 4.00E-95 | Solyc04g011430.2 | **Solyc05g054540.2** | 0.0563 | 0.3343 | 0.168412 |
| 310 | 3015 | 5.00E-111 | Solyc07g062570.2 | **Solyc10g007260.2** | 0.0057 | 0.5703 | 0.009995 |
| 328 | 280 | 2.00E-111 | **Solyc07g053960.2** | Solyc12g013820.1 | 0.5189 | 1.6416 | 0.316094 |
| 116 | 958 | 2.00E-100 | Solyc02g078210.2 | **Solyc07g053960.2** | 0.5584 | 3.1307 | 0.178363 |
| 72 | 234 | 1.00E-105 | **Solyc01g095490.2** | Solyc12g056100.1 | 0 | 0 | 0 |
| 58 | 1751 | 1.00E-108 | **Solyc01g095490.2** | Solyc10g011740.2 | 0.0117 | 0.7122 | 0.016428 |
| 27 | 716 | 5.00E-106 | **Solyc01g095490.2** | Solyc05g050230.2 | 0.0266 | 0.5492 | 0.048434 |
| 16 | 364 | 3.00E-107 | **Solyc01g095490.2** | Solyc03g007470.2 | 0.0244 | 1.9591 | 0.012455 |
| 243 | 412 | 2.00E-107 | **Solyc05g050230.2** | Solyc10g011740.2 | 0.0146 | 0.6703 | 0.021781 |
| 146 | 230 | 3.00E-106 | **Solyc03g007470.2** | Solyc05g050230.2 | 0.0266 | 2.073 | 0.012832 |
| 186 | 264 | 5.00E-108 | **Solyc03g007470.2** | Solyc10g011740.2 | 0 | 0 | 0 |
| 133 | 221 | 6.00E-87 | Solyc02g078210.2 | **Solyc12g013820.1** | 0.9276 | 2.0818 | 0.445576 |
| 100 | 1872 | 2.00E-99 | Solyc02g084760.2 | **Solyc04g080810.2** | 0.0608 | 1.8528 | 0.032815 |
| 91 | 572 | 3.00E-109 | **Solyc02g087750.2** | Solyc03g113100.2 | 0.0324 | 2.1438 | 0.015113 |

**Supplementary Table S5:** The Ka and Ks relation of 59 E2 ubiquitin conjugating enzymes along with synteny block information.

**
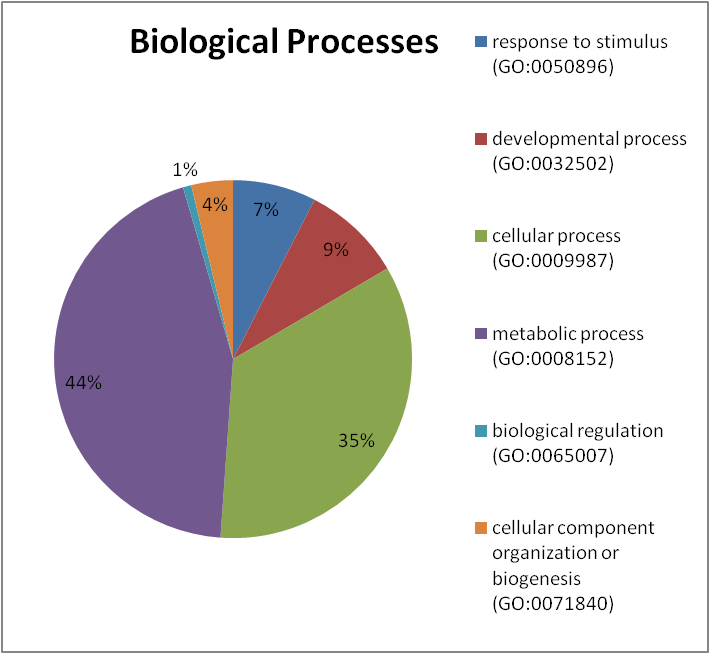
**

**Supplementary Figure S6:** The chart representing Gene Ontology (Biological processes) of 59 E2 enzymes.

**
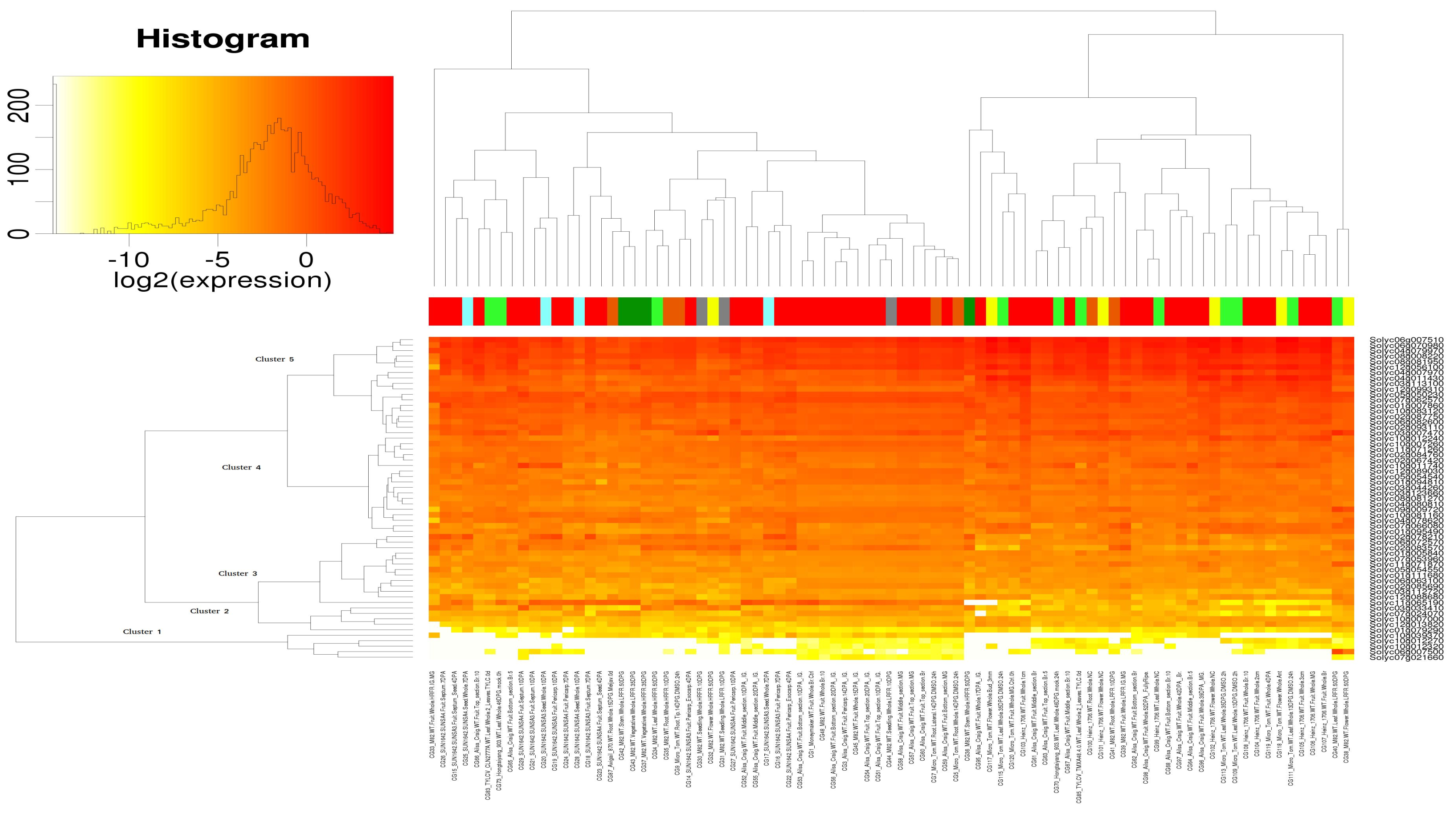
Supplementary Figure S7 :** The gene expression level of 59 tomato E2 enzyme sequences in seed, seedling, root, vegetative, leaf, flower and fruit tissue of Micro Tom, Heinz 1706, M82, Moneymaker, SUN1642, and Alisa Craig cultivars of tomato is illustrated . The normalized gene expression level is represented by a color scale histogram. Cluster 1 and 2 with maximum genes, were highly expressed in most of the conditions compared to cluster 3, 4 and 5.

**
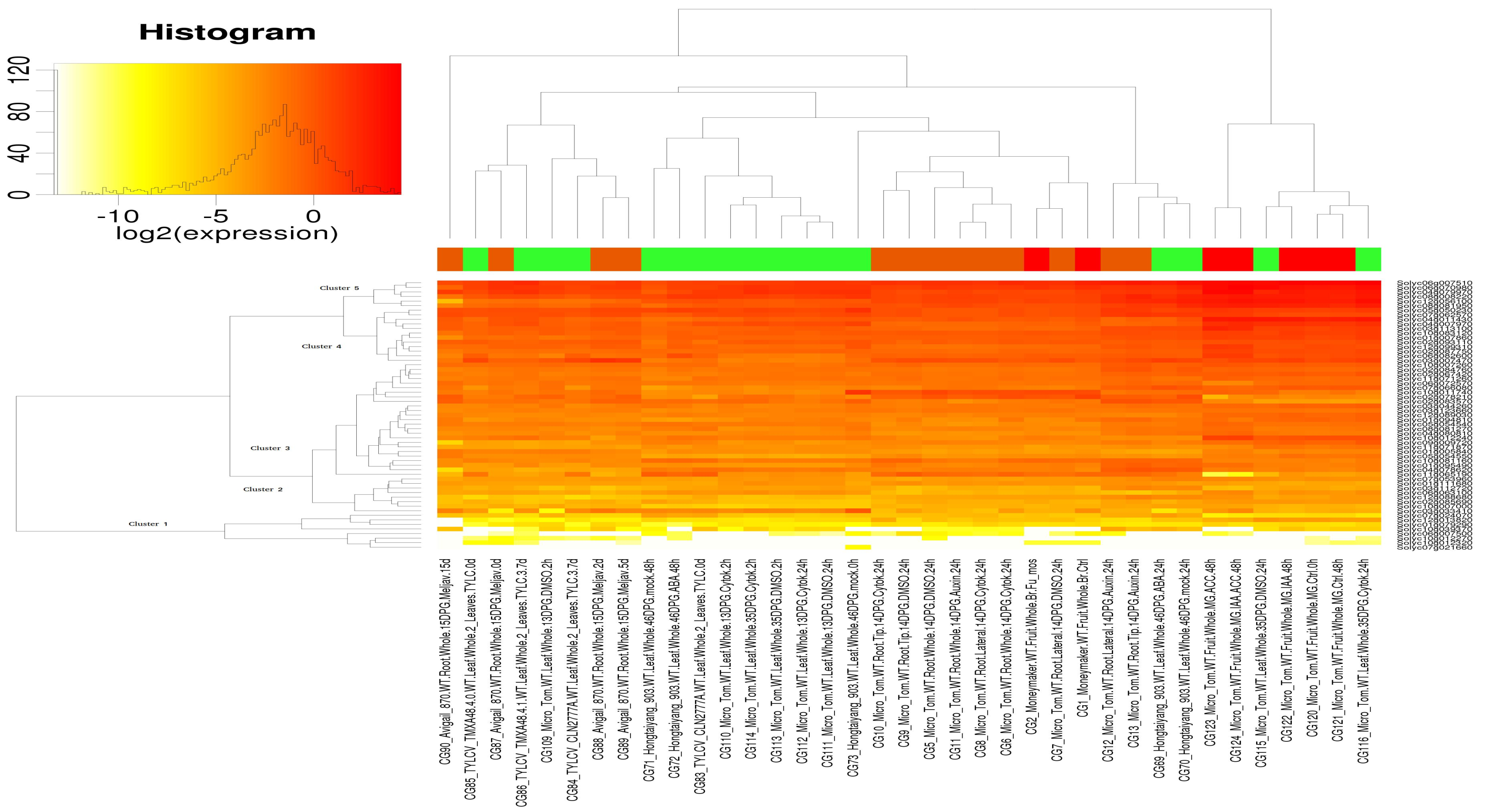
**

**Supplementary Figure S8:** The gene expression level of 59 tomato E2 enzyme sequences during hormone treatment and pathogen infection in root, leaf and fruit tissues of Micro Tom, Avigail, Hongtaiyamg, Moneymaker cultivars of tomato. The normalized gene expression level is represented by a color scale histogram.
